# Interactions of AMTN, ODAM and SCPPPQ1 proteins of a specialized basal lamina that attaches epithelial cells to tooth mineral

**DOI:** 10.1038/srep46683

**Published:** 2017-04-24

**Authors:** Aurélien Fouillen, Juliana Dos Santos Neves, Charline Mary, Jean-Daniel Castonguay, Pierre Moffatt, Christian Baron, Antonio Nanci

**Affiliations:** 1 Laboratory for the Study of Calcified Tissues and Biomaterials, Department of Stomatology, Faculty of Dental medicine Université de Montréal, Montréal, Québec, Canada; 2 Department of Biochemistry and Molecular Medicine, Faculty of Medicine, Université de Montréal, Québec, Canada; 3 Shriners Hospital for Children, Montréal, Québec, Canada

## Abstract

A specialized basal lamina (sBL) mediates adhesion of certain epithelial cells to the tooth. It is distinct because it does not contain collagens type IV and VII, is enriched in laminin-332, and includes three novel constituents called amelotin (AMTN), odontogenic ameloblast-associated (ODAM), and secretory calcium-binding phosphoprotein proline-glutamine rich 1 (SCPPPQ1). The objective of this study was to clarify the structural organization of the sBL. Fluorescence and immunogold labeling showed that the three proteins co-localize. Quantitative analysis of the relative position of gold particles on the sBL demonstrates that the distribution of ODAM is skewed towards the cell while that of AMTN and SCPPPQ1 tends towards the tooth surface. Bacterial two-hybrid analysis and co-immunoprecipitation, gel filtration of purified proteins and transmission electron and atomic force microscopies highlight the propensity of AMTN, ODAM, and SCPPPQ1 to interact with and among themselves and form supramolecular aggregates. These data suggest that AMTN, ODAM and SCPPPQ1 participate in structuring an extracellular matrix with the distinctive capacity of attaching epithelial cells to mineralized surfaces. This unique feature is particularly relevant for the adhesion of gingival epithelial cells to the tooth surface, which forms a protective seal that is the first line of defense against bacterial invasion.

Basal laminae (BLs) act as an interface between epithelial cells and the underlying connective tissue, and comprise collagen types IV and VII, proteoglycans, and glycoproteins such as laminins^1^,^2^,^3^. They provide tissue boundaries and structural support, influence cellular organisation, serve as physical barriers and mediate intracellular signaling cascades^4^. In the tooth, a specialized BL (sBL) binds epithelial cells to mineralized surfaces rather than connective tissue, thereby creating secluded environments that are critical for mineralization and for protection of the tooth supporting tissues from the aggressive oral environment^5^,^6^.

The epithelial enamel organ (EO) is associated with the formation of the hardest mineralized matrix in the human body, tooth enamel. The EO is comprised of cells called ameloblasts that have a complex life cycle with distinct stages. During the pre-secretory stage, a typical BL separates differentiating ameloblasts from differentiating odontoblasts^7^. This BL is removed just before progressing into the subsequent secretory stage during which the active deposition of enamel proteins guides the formation, organization and partial mineralization of the entire enamel layer. Next, at the onset of the maturation stage, ameloblasts deposit a sBL along their apical surface. The sBL attaches the apical surface of ameloblasts to the maturing enamel surface forming a confined space for enzymatic degradation of proteins that will permit enamel crystal expansion in thickness and width^8^. This sBL has an atypical composition; it does not contain γ1 chain-containing laminins and collagen types IV and VII^2^,^3^, but is enriched in laminin-332 (LM-332)^9^,^10^,^11^. Once enamel has fully completed its mineralization, the tooth erupts and part of the EO covering the tooth crown fuses with the oral epithelium. This fusion results in the formation of a structure called junctional epithelium (JE), which adheres to the tooth surface through a sBL similar in composition to that found in the maturation stage EO. The JE is a specialized portion of the gingiva that seals off the tooth supporting tissues from the aggressive oral environment. As such, it forms an epithelial barrier against bacterial invasion and thus represents the first line of defense against periodontal disease (PD)^6^,^12^. The mechanisms by which the JE adheres to the tooth surface, via the sBL, are still poorly understood.

Transcriptomic screens of rat and mice EOs have led to the discovery of three genes encoding novel proteins named amelotin (AMTN), odontogenic ameloblast-associated (ODAM), and secretory calcium-binding phosphoprotein proline-glutamine rich 1 (SCPPPQ1)^13^,^14^,^15^,^16^. These genes are members of the secretory calcium-binding phosphoprotein (SCPP) gene cluster and the encoded proteins are evolutionarily related to SPARC, a well-known matricellular protein that participates in the assembly of typical BLs^17^,^18^,^19^. Proteins from this cluster stabilize calcium and phosphate ions in tissue fluids and regulate their deposition in the extracellular matrix, although this has not been formally determined for AMTN, ODAM and SCPPPQ1. They are produced by maturation stage ameloblasts and JE cells, and high-resolution immunogold analysis revealed that the three proteins localize to the sBL^16^,^20^,^21^. As such, they likely participate in structuring the supramolecular architecture of the sBL. Since AMTN, ODAM and SCPPPQ1 are unique to the sBL, they appear crucial to structuring this special extracellular matrix and to mediating the adhesion of epithelial cells to tooth surfaces. In support of this notion, recent reports have shown JE detachment in ODAM-KO mice and loss of integrity of the maturation stage sBL in a transgenic mouse model expressing human laminin-γ2^22^,^23^,^24^.

A yeast two-hybrid (YTH) analysis has demonstrated that bovine ODAM and AMTN proteins interact but nothing is known about the behaviour of SCPPPQ1^25^. There is also no quantitative evaluation of their interacting capacity nor any structural information on the resulting aggregates. Such information is a prelude for understanding the mechanisms that are implicated in structuring the sBL and its adhesion to mineralized surfaces.

The objective of our study was to analyse the relative distribution of AMTN, ODAM and SCPPPQ1 in the sBL, the interactions of their human forms, and the structure of the resulting assemblies. Bacterial two-hybrid analysis (BTH) and both transmission electron (TEM) and atomic force (AFM) microscopy demonstrate the propensity of all 3 molecules to interact as homo- as well as heteromultimers. Immunofluorescence, immunogold labeling and Nearest Neighbour Analysis suggest that the distribution of ODAM within the sBL is skewed towards the cell, while AMTN and SCPPPQ1 tend to localize towards the tooth surface. Altogether, these data suggest that AMTN, ODAM, and SCPPPQ1 interact to structure a specialized macromolecular complex capable of binding on one side to epithelial cells and on the other to tooth mineral.

## Results

### Spatial distribution of AMTN, ODAM, and SCPPPQ1 in the sBL

**Figure 1:**
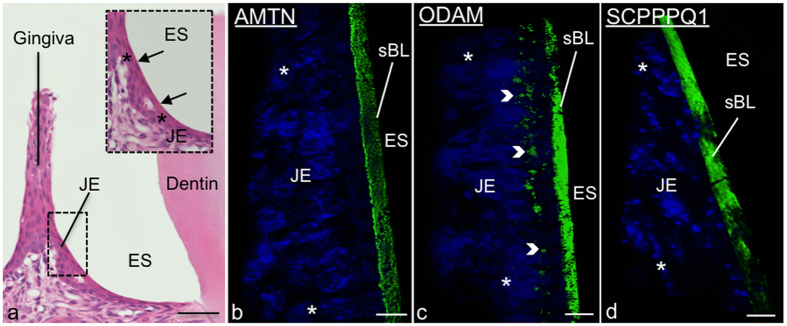
AMTN, ODAM and SCPPPQ1 localize at the cell-tooth interface. (**a**) Micrograph from a histological section stained with hematoxylin and eosin showing apposition of the junctional epithelium (JE) to the enamel, seen here as a space (ES) following decalcification. The inset is a higher magnification of the JE cells (*). The arrows point to the position of the associated specialized basal lamina (sBL). (**b**–**d**) Immunofluorescence preparations for AMTN, ODAM and SCPPPQ1 imaged by Structured Illumination Microscopy (SIM) indicate that the three proteins reside in the sBL. ODAM also exhibits a cell-associated labeling (arrowheads). Proteins stained in green, nuclei in blue using DAPI. Scale bars = 10 μm.

**Figure 2:**
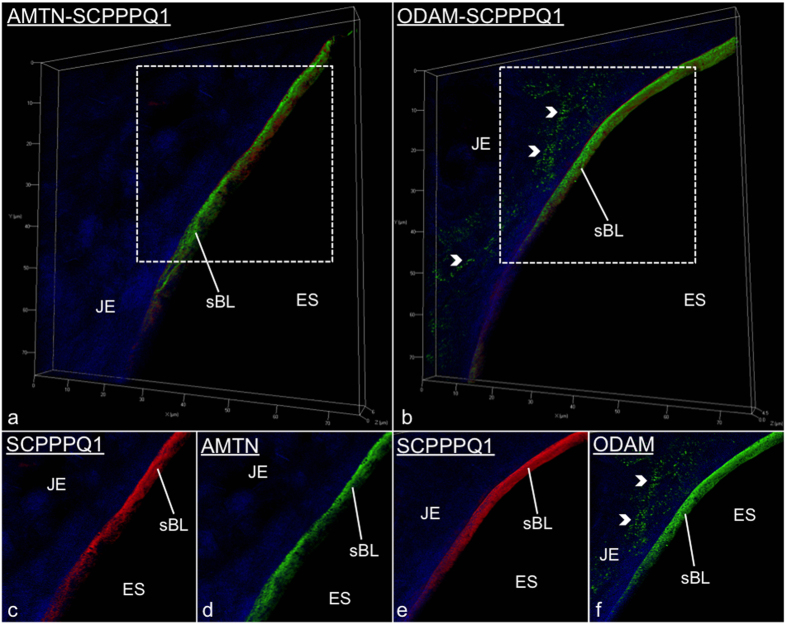
Immunofluorescence labeling for AMTN, ODAM and SCPPPQ1 visualized in junctional epithelium (JE) preparations by Structured Illumination Microscopy. (**a** and **b)** Illustrate dual-labelled preparations. (**c**–**f)** Are the corresponding single channel images at higher magnification. These dual preparations validate that the proteins co-localize at the cell-tooth interface where the specialized basal lamina (sBL) is found. AMTN/ODAM in green; SCPPPQ1 in red; nuclei in blue using DAPI.

**Figure 3:**
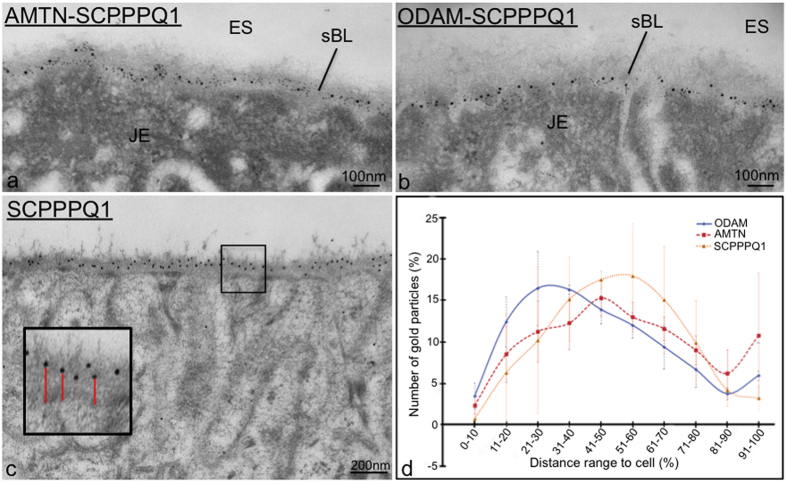
High resolution immunogold labeling demonstrates that AMTN, ODAM and SCPPPQ1 distribute differentially across the specialized basal lamina (sBL). (**a**,**b**) As exemplified here with the maturation stage enamel organ (EO), dual labeling evidences with high resolution the co-localisation of the three proteins in the specialized basal lamina (sBL). (**c**,**d**) Single immunogold preparations; Quantification of the distribution of particles relative to the cell surface reveals that the three proteins distribute differentially across the sBL. AMTN/ODAM 10 nm gold particles; SCPPPQ1 20 nm gold particles.

The JE (Fig. 1a) and EO consist of a multiple cell layer bordering enamel, seen as a space following decalcification. As for all basal laminae, the sBL is not readily visible in conventional histological preparations and must be revealed by histochemical methods such as immunostaining. Here we have applied structured illumination microscopy (SIM) to visualize the three-dimensional localization of AMTN, ODAM, and SCPPPQ1 at the cell-tooth interface (Fig. 1b–d). Dual fluorescence and colloidal gold immunolabelings were then used to better define their relationship within the sBL. The use of fixation and blocking steps between antibody incubations make it possible to achieve dual labelings on the same face of the tissue section using antibodies from the same species^26^. This approach shows that the three proteins undisputedly co-localize in the sBL of JE (Fig. 2) and of maturation stage ameloblasts (Fig. 3a,b). Nearest-Neighbour Analysis (NNA) was carried on single immunogold labeling preparations to obtain quantitative information on the relative distribution of each protein within the sBL. This powerful analytical method is used to determine the spatial distribution of objects relative to a reference point, in our case the position of colloidal gold particles with respect to the cell surface (Fig. 3c). NNA revealed that the distribution of ODAM within the sBL is skewed toward the cell, while AMTN and particularly the smaller molecular weight SCPPPQ1 tend to localize more towards the tooth surface (Fig. 3d).

### Analysis of the secondary structure of AMTN and ODAM

To evaluate the secondary structures of recombinant human ODAM (hODAM) and human AMTN (hAMTN) overproduced and purified from *E. coli,* we have used circular dichroism (CD) spectroscopy^27^. CD spectra were determined across a range of concentrations, with representative spectra of 5 μM hAMTN and 10 μM hODAM solutions shown here (Fig. S1). Analysis of these spectra using the CDSSTR method^28^ from Dichroweb^29^ indicated that hAMTN is comprised of 40% β-sheet, 12% turns, and 44% largely disordered regions. hODAM is also largely disordered (47%) and is comprised of 35% β-sheets and 13% turns. Interestingly, combining hODAM and hAMTN reduced the degree of disorder while significantly increasing the amount of α-helices (54%), suggesting conformational changes upon mixing and interactions between these proteins (Fig. S1).

### Protein-protein interaction studies

**Figure 4:**
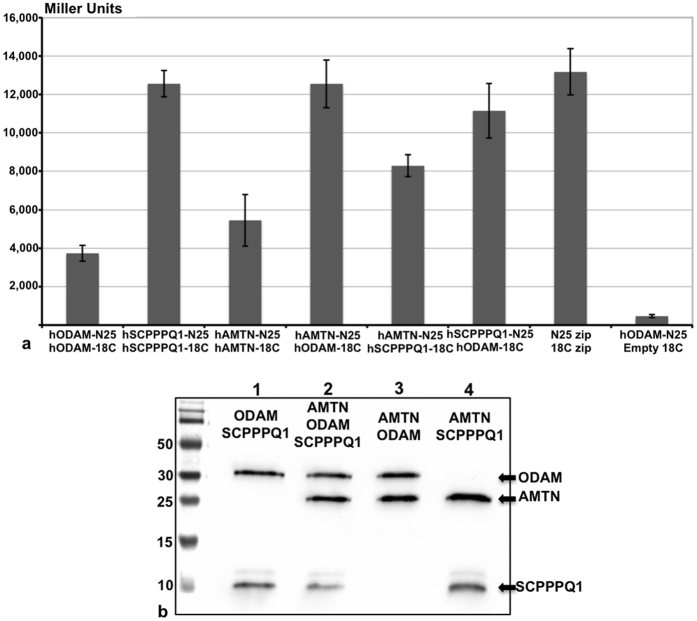
Interaction analysis between AMTN, ODAM and SCPPPQ1. (**a**) Bacterial two-hybrid analysis between human proteins after fusions to the PKNT25 and PUT18C domains of adenylate cyclase. All 3 proteins have the propensity to interact, with AMTN-ODAM and SCPPPQ1-ODAM interactions showing the highest level of interaction. Positive control = PUT18Czip +PKNT25zip; negative control = hSCPPPQ1-PKNT25 + empty PUT18C vector. (**b**) These interactions are confirmed by co-immunoprecipitation followed by Western blotting.

Given their close proximity within the sBL and the general behaviour of flexible proteins, we have examined the propensity of AMTN, ODAM and SCPPPQ1 to interact. A number of complementary approaches were used here to achieve biochemical, visual, and quantitative corroborative data. The BTH assay was applied to assess interaction in a quantitative *in vivo* model. Different combinations of fusions of the three proteins to domains of adenylate cyclase were co-expressed in adenylate cyclase-negative *E.coli* BTH101 cells. Interactions leading to restoration of adenylate cyclase enzyme activity were measured using cAMP-inducible β-galactosidase activity. All combinations tested yielded enzymatic activity albeit at variable levels. Irrespectively, as illustrated by the histogram in Fig. 4a, hODAM-hSCPPPQ1 and hODAM-hAMTN exhibit a stronger potential for interaction while hAMTN-hSCPPPQ1 show a lesser one. Noteworthy, hSCPPPQ1 shows the highest propensity for self-interaction among the three proteins. To evaluate the potential that interactions differ between species, we have also carried out BTH analysis with the rat homologs that have ~20% difference in amino acid composition^16^,^30^,^31^. Essentially, no qualitative difference of the capacity to interact was observed when homologous or heterologous interactions between rat and human proteins were tested, as exemplified by AMTN (Fig. S2). Interactions revealed by two-hybrid assays must be confirmed and validated using an alternative approach. To this effect, we also performed co-immunoprecipitation on *E. coli* BL21(DE3)-star lysates used for the co-expression of the proteins. The three proteins were pulled down with each of the three specific antisera, hence validating our BTH results (Fig. 4b).

### Gel filtration characterization of AMTN and ODAM

To further characterize the supramolecular assemblies formed by AMTN and ODAM proteins, we have over-expressed the full-length recombinant human proteins lacking their N-terminal signal sequence in *E. coli* BL21(DE3)-star and purified them by Ni^2+^ affinity chromatography. The elution profile of size exclusion chromatography revealed that recombinant hODAM (~32 kDa) was found in the high molecular weight fraction of ~400 kDa (Fig. S3A), equivalent to an aggregate of 12–13 subunits. hAMTN (~21 kDa) formed aggregates having a molecular mass of around 110 kDa (Fig. S3B), equivalent to a pentamer. SDS-PAGE confirmed that proteins in the gel filtration fractions of hODAM and hAMTN correspond to expected molecular masses and demonstrate the purity of the samples (Fig. S3C and D). Study of recombinant rat homologs revealed similar gel filtration profiles, demonstrating that protein behaviour is not species-dependent (data not shown).

### Structural characterization of supramolecular complexes formed by AMTN, ODAM and SCPPPQ1

**Figure 5:**
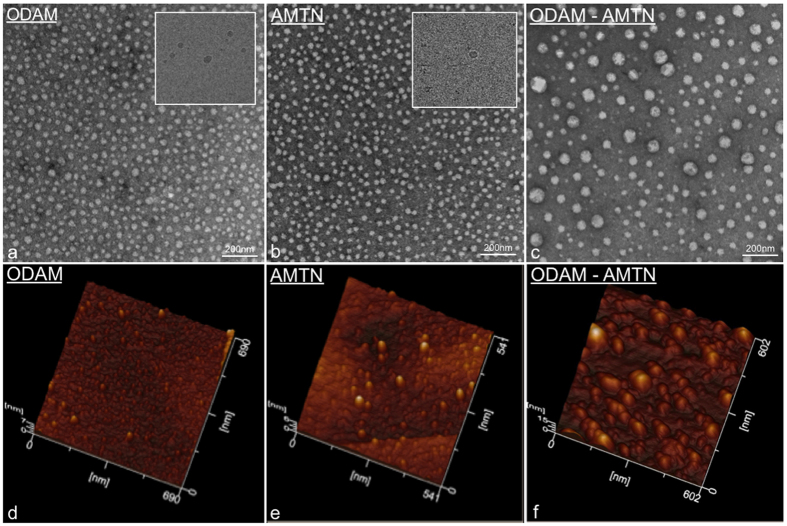
Visual characterization of the aggregation behaviour of AMTN and ODAM. (**a–c**) Transmission electron microscopy (TEM) of negative stain and (**d–f**) atomic force microscopy preparations of purified AMTN and ODAM demonstrate the propensity of the proteins to self-interact into globular complexes. Mixing the two proteins together generates larger complexes suggesting heterologous interactions. (Insets in **a**,**b**) Observation of the complexes under native conditions by cryo-TEM confirms that they do not result from preparation artefacts.

**Figure 6:**
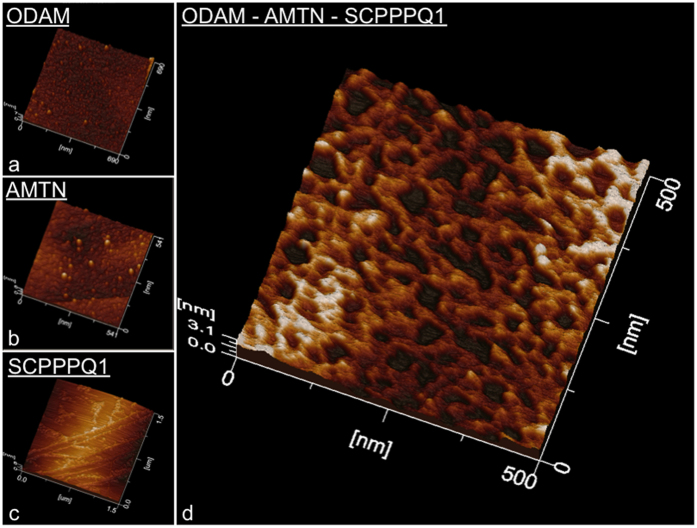
Atomic force microscope characterization of AMTN, ODAM and SCPPPQ1 proteins. (**a**–**c**) The discrete profiles formed individually by each protein differ dramatically from (**d**) the intricate network formed created when they are mixed together.

Negative staining TEM and AFM were applied to visualize the shape of recombinant hODAM and hAMTN proteins collected in the gel filtration fractions. In each case using TEM, globular structures were observed (Fig. 5a,b), with diameters ranging from 18.6 to 27.4 nm with a mean of 22.31 ± 3.5 nm (n = 50) for hAMTN, and from 24.3 to 36.9 nm with a mean of 30.26 ± 3.8 nm (n = 50) for hODAM. Interestingly, when both proteins were combined, larger globular structures formed, the diameters of which now ranged from 19.8 to 74.1 nm with a mean of 44.08 ± 12.13 nm (n = 50) (Fig. 5c). Cryo-TEM imaging of the proteins in their native state (Fig. 5a,b Insets) and AFM that relies on protein interaction with the Highly Ordered Pyrolytic Graphic (HOPG) substrate (Fig. 5d–f) validate the globular nature of aggregates. Because SCPPPQ1 requires urea to be solubilized and AFM tolerates the presence of some denaturing buffer, SCPPPQ1 alone and the mix of the three proteins were observed by AFM. By itself SCPPPQ1 formed linear profiles while, in the presence of both AMTN and ODAM, it generated a porous network (Fig. 6).

## Discussion

Three proteins -AMTN, ODAM, and SCPPPQ1- have been identified as novel constituents of the sBL that mediates attachment of epithelial cells to tooth surfaces^15^,^16^,^31^. As knowledge of physical behaviour of proteins is fundamental in defining their role and function, we have applied a complement of biochemical and imaging approaches to better define their distribution within the sBL, their propensity to interact and form supramolecular aggregates, and the structure of resulting molecular assemblies.

### ODAM, AMTN and SCPPPQ1 are differential distributed across the sBL

Our results show that AMTN, ODAM, and SCPPPQ1 assume a differential distribution within the sBL. The preferential presence of ODAM near the cell surface suggests that it participates in defining the face of the sBL that mediates its anchoring to the epithelial cells (ameloblasts, gingival cells). On the other hand, the tendency of AMTN and SCPPPQ1 to predominate on the opposite side of the sBL places them in a position to potentially play a role in binding of the sBL to the tooth surface. However, the fact that (a) the ODAM-KO does not show a phenotype during the maturation stage and only shows a partial delamination of the JE^22^, and that (b) the AMTN-KO mice show no detachment of both maturation stage ameloblasts and JE cells^32^ indicate that the situation is more complex and that there may be other bridging molecules implicated in the process. In the case of AMTN there may be compensation by SCPPPQ1. In fact, NNA suggests that it is closest to the enamel surface positioning SCPPPQ1 as a potential direct or indirect mediator for adhesion of the sBL to mineral.

### AMTN, ODAM and SCPPPQ1 exhibit propensity to interact

BTH has been intensively used to analyze protein interactions in multimolecular assemblies^33^. As compared to a previous YTH study that solely relied on a qualitative colorimetric evaluation of yeast cultures^25^, we have applied a quantifiable assay to characterize the interactions^34^. All three epithelial proteins interact, with ODAM showing a strong affinity for its two sister proteins, AMTN and SCPPPQ1. In comparison with AMTN and ODAM, the higher affinity of SCPPPQ1 for self-interaction observed by BTH is consistent with its tendency to aggregate during purification in non-denaturing conditions^16^. ODAM and AMTN are intrinsically disordered proteins. The presence of large disordered regions that impart structural flexibility is a trait shared by members of the SCPP family^35^. It is well established that intrinsically-disordered portions of proteins are considered as flexible linkers that freely rotate and recruit surrounding partners to form multidomain complexes^36^. This may explain why mixing hODAM and hAMTN decreases the degree of disorder and results in the formation of α-helices, further demonstrating that the two proteins interact. In addition, no qualitative difference of the capacity to interact was observed when homologous or heterologous interactions between rat and human proteins were tested, as exemplified by AMTN (Fig. S2), suggesting that interaction sites reside in the conserved amino acid portions of these proteins. Both BTH and coIP studies unequivocally demonstrate the propensity of AMTN, ODAM, and SCPPPQ1, whether of rat or human origin, to form homo- as well as hetero-multimers. In addition to demonstrating that all three proteins interact, these quantitative enzymatic evaluations are essential for follow-up BTH studies with constructs modified by site-directed mutagenesis of potential interaction sites that will be identified by phage display analyses^37^. Such studies will allow us to identify motifs or specific amino acids responsible for the interactions.

### Structural evidence that the proteins form supramolecular complexes

Consistent with the BTH results, TEM and AFM data provide structural evidence that AMTN and ODAM individually have the potential to form globular self-aggregates under the condition used. The discrepancy in size observed between TEM and size exclusion chromatography may relate to the fact that gel filtration is sensitive to conformation. Independently of the conditions used for sample preparation, AMTN and ODAM homo-oligomers also interact *in vitro* to form larger hetero-oligomers. Preparation of samples for negative stain TEM or AFM requires drying of the samples, during which proteins may artefactually aggregate as liquid is removed. While more extensive analyses will be required for quantitative evaluation of size distribution, cryo-TEM results validate the tendency of hAMTN and hODAM to self-assemble into globular forms in their native state, and rule out the possibility that this results from drying. Also, the larger aggregates observed *in vitro* in the hAMTN-hODAM mixtures are unlikely to occur in the sBL because of space limitations and therefore there must be mechanisms to limit the aggregative capacity *in vivo*. Interestingly, SCPPPQ1 alone forms linear profiles but mixing with its two sister proteins results in a porous supramolecular network. Such networks have been described for conventional BLs^38^. Therefore, while SCPPPQ1 might have an affinity for the tooth surface, it may also play a bridging and stabilizing role in the sBL.

**Figure 7:**
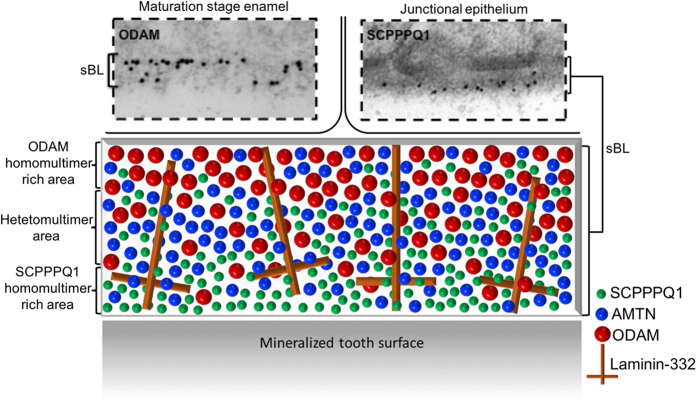
Schematic model of the distribution of AMTN, ODAM and SCPPPQ1 within the supramolecular organisation of the specialized basal lamina (sBL). The two boxed above in images are representative immunogold labelings for ODAM (skewed toward the cells) and SCPPPQ1 (skewed toward the tooth surface) over the sBL from the maturation stage enamel organ and the junctional epithelium. The model attempts to integrate the observed differential distribution of the three proteins within a laminin-332 background whose arrangement within the unique extracellular matrix constituted by the sBL remains to be clarified.

The abundance of Laminin-332 in the sBL^9^,^10^ may actually provide a background for ordered supramolecular interactions with AMTN, ODAM and/or SCPPPQ1 that determines its integrity. The HOPG substrate used for AFM may play a similar role *in vitro*. In a recent report, we have shown that the sBL is disrupted in a mouse model expressing human Lam-γ2^23^. Substitution of mouse *LAMC2* by its human form seems to interfere with these interactions, resulting in the dispersion of AMTN, ODAM and SCPPPQ1. On the basis of what is known today, we provide here a ‘tentative’ representation for the integration of AMTN, ODAM and SCPPPQ1 within the supramolecular organisation of the sBL that reflects their localisation and their propensity to aggregate, and that takes into account the Laminin-332 background (Fig. 7).

## Conclusion

The spatial arrangement of molecules, their structural organization, as well as their interactive behaviour provide critical information on their functions. In this context, our objective was to characterize the structural distribution and organization of AMTN, ODAM and SCPPPQ1, as well as their propensity for homologous and heterologous interactions. They differentially accumulate throughout the sBL to participate in the supramolecular assembly of an extracellular matrix with the unique capacity to bind epithelial cells to mineralized surfaces. A better understanding of the supramolecular architecture of the sBL and of the molecules that determine the interfacial biology between the JE and the tooth surface is expected to pave the way for innovative treatments for PD. In addition, knowledge of the molecular behaviour of ODAM is particularly relevant because it has also been associated with epithelial cancers in tissues of origin that do not normally express the molecule^39^.

## Methods

All animal procedures were approved by the Comité de déontologie de l’expérimentation sur les animaux of Université de Montréal and all methods were performed in accordance with their guidelines and regulations.

### Tissue processing for light and electron microscopy

Six-month old wild type mice were anesthetized with 20% chloral hydrate solution (0.4 mg/g body weight; Fisher Scientific, Whitby, ON, Canada) and sacrificed by perfusion through the left ventricle with Ringer’s lactate (Abbott Laboratories; Montreal, QC, Canada) for 30 s, followed by a fixative solution consisting of 4% paraformaldehyde (BDH; Toronto, ON, Canada) and 0.1% glutaraldehyde (Electron Microscopy Sciences; Washington, PA) in 0.1 M phosphate buffer (PB, pH 7.2) for 10 min. Mandibles and maxillae were dissected, and specimens were immersed in the same fixative solution for 3 h at 4 °C. The samples were rinsed in 0.1 M PB (pH 7.2) overnight at 4 °C. Specimens were decalcified with 4.13% disodium ethylenediamine tetra-acetic acid (EDTA, Fisher Scientific) for 21 days and were then washed for 24 h in 0.1 M PB (pH 7.2).

Some decalcified specimens were processed for paraffin embedding. Five μm thick sections were prepared with a Leica RM2155 microtome (Leica Microsystems Canada, Richmond Hill, ON, Canada) and mounted on Superfrost^®^/Plus slides (Fisher Scientific) and stained with hematoxylin and eosin for morphological analyses or processed for immunofluorescence labeling. Other decalcified specimens were post-fixed with potassium ferrocyanide-reduced osmium tetroxide and then processed for embedding in LR White resin (London Resin Company; Berkshire, UK) as previously reported^40^. Ultrathin 80–100 nm sections were cut with a diamond knife and transferred onto Formvar^®^-coated (polyvinyl formate) 200-mesh nickel grids for immunogold labeling.

### Immunofluorescence

Briefly, deparaffinized sections were blocked in 0.01 M phosphate buffered saline (PBS, pH 7.2) containing 5% skim milk (Carnation; Nestle, Don Mills, ON, Canada) for 1 h. They were then incubated for 3 h with primary antibodies for rat ODAM (1:10000)^41^, rat AMTN (1:2000)^15^, or rat SCPPPQ1 (1:2000)^16^, followed by a secondary goat anti-rabbit AlexaFluor 488 or 562 antibodies (1:500, 1 h, Life Technologies™, Mississauga, ON, Canada). Following each step, the slides were washed 3 times for 10 min each with PBS-Tween (0.05% (v/v)). For dual immunolabeling, following a first round of immunofluorescence, the samples were fixed using 0.01% glutaradehyde before performing a second round of immunofluorescence. Sections were then mounted in ProLong Gold containing DAPI (Life Technologies™) in order to stain nuclei. Negative controls included omission of primary antibody and incubation with pre-immune serum. Also, controls for the antibodies used have been previously reported^16^,^30^,^31^. Sections were examined under structured illumination microscopy using an Elyra PS1 microscope (Carl Zeiss, Oberkochen, Germany). Z-stack volumes were acquired using the Structural Illumination Method (SIM) and reconstructed using the Zen Black edition software.

### Post-embedding colloidal gold immunocytochemistry

Ultrathin sections of osmicated samples were first treated with an aqueous solution of 5% sodium metaperiodate^42^ for 45 min and washed with distilled water. Grids were then floated on a drop of 1% ovalbumin in 0.01 M phosphate buffered saline (PBS, pH 7.2) for 15 min for blocking of unspecific binding and then transferred onto a drop of primary antibody for 1 h (anti-SCPPPQ1 1:500; anti-ODAM 1:800, anti-AMTN 1:800). Following incubation with primary antibody, the grids were rinsed with PBS and placed again in blocking solution for 15 min. The sites of antibody-antigen binding were then revealed by floating the grids on a drop of the protein A-20 nm colloidal gold complex for 30 min. For dual immunolabeling, following a first round of labeling using anti-AMTN or anti-ODAM revealed with 10 nm colloidal gold, the samples were floated on 0.01% glutaradehyde before performing a second round of labeling using anti-SCPPPQ1 revealed with 20 nm colloidal gold. Finally, the grids were washed with PBS followed by distilled water. All steps were carried out at room temperature. Controls consisted of incubations with protein A-gold alone. Sections were then stained with uranyl acetate and lead citrate and examined in an FEI Tecnai 12 (Eindhoven, The Netherlands) transmission electron microscope operating at 80 kV.

For nearest neighbour analysis, a minimum of 10 pictures were taken of each maturation region (early-mid, late) of an incisor (n = 6) for each protein studied. The distance between gold particle over the BL and the cell membrane was measured using AnalySIS_ software (Soft Imaging System GmbH, Lakewood, CO). The percentage correspond to the distance measured for each picture, where values 0–10% closer to the cells and 90–100% near the enamel surface. The data particle counts within each distance interval were found to follow a normal distribution and were analyzed using a t test (QI macros, Excel SPC Software); the level of significance was set at p < 0,05.

### Cloning procedures

Truncated versions of *odam, amtn,* and *scpppq1* genes lacking regions encoding the predicted N-terminal signal sequence were PCR-amplified from human cDNA sequences using primers listed in Table S4. PCR products were cloned into pUT18C or pKNT25 vectors used for BTH analysis or into the vector pHT for co-immunoprecipitation or purification studies^43^. The recombinant pHT plasmids allow to produce recombinant proteins with an in-frame N-terminal hexahistidyl-tag (His-tag) and TEV protease cleavage site. *Escherichia coli* strains XL-1 Blue were used as hosts for cloning.

### BTH

Interactions between the 3 proteins were assessed *in vivo* using the Bacterial Two-Hybrid system (BTH). Genes of interest were fused to DNA sequences encoding the T18 and T25 fragments of the catalytic domain of *Bordetella pertussis* adenylate cyclase (AC) and were co-expressed in *E.coli* BTH101 AC (cya) deficient cells. Interactions were detected by functional complementation between the two catalytic AC fragments, which leads to cAMP/ß-galactosidase production. These were detected using firstly a white/blue colonies assay using X-gal (5-bromo-4-chloro-3-indolyl-ß-D-galactopyranoside) substrate and then by quantification using ortho-nitrophenyl-ß-D-galactopyranoside (ONPG) substrate.

### CoIP

One ml of *E. coli* BL21(DE3)-star strain co-expressing two or three recombinant protein pHT vectors was collected by centrifugation at 7,000 g for 15 min. The pellet was re-suspended in 100 μl lysis buffer (50 mM HEPES, 5 mM MgCl_2_, 25% sucrose, 1% Triton X-100, 1 mg/ml Lysozyme pH 8) and incubated on ice for 1 h. The suspension was then centrifuged at 12,000 g for 30 min. Antibody (anti-AMTN 1:1000 or anti-ODAM 1:1000) was added to the supernatant and incubated for 2 h at 4 °C. Protein A-Sepharose slurry (Pierce Co-Immunoprecipitation kit, ThermoFisher scientific) was subsequently added, followed by incubation for 2 h at 4 °C. Nonspecific binding proteins were removed by five successive rinses with phosphate buffered saline (PBS). The Protein A-Sepharose was finally eluted with glycine solution (0.1 M; pH 1.8). The eluate was collected and analyzed using sodium dodecyl sulfate-polyacrylamide gel electrophoresis (SDS-PAGE) followed by Western blot using anti-His-tag antibodies (1:200).

### Protein overexpression and purification

BL21(DE3)-star cells containing either pHT*hAmtn* or pHT*hOdam* were grown at 37 °C and 250 rpm to an O.D_600_ around 0.6, and protein expression was induced with 0.1 mM isopropyl-ß-D-thiogalactoside (IPTG) for 5 h at 30 °C and 250 rpm. Bacterial cells were harvested, suspended in Equilibration buffer (50 mM Na_2_HPO_4_, 150 mM NaCl, 10 mM Imidazole, pH 7) at 4 °C, and sonicated six times 15 s between 15 s ice incubations. Lysates were centrifuged at 13,400 g and the 6His-tagged protein in the supernatant was bound on nickel-nitriloacetic acid (Ni-NTA)-agarose affinity resin (QIAGEN) at room temperature. After washing the resin with 10 volumes of Binding buffer (50 mM Na_2_HPO_4_, 300 mM NaCl, 20 mM Imidazole, pH 7), proteins were eluted with Elution buffer (50 mM Na_2_HPO_4_, 300 mM NaCl, 300 mM Imidazole, pH 7). Collected fractions were assessed for protein content using the Bradford assay and analyzed by SDS–PAGE and Coomassie blue staining. Chosen fractions were then dialyzed into TEV buffer (25 mM Na_2_HPO_4_, 125 mM NaCl, 5 mM DTT, pH 7.4) and had their N-terminal His-tag cleaved using His-tagged TEV protease (Sigma-Aldrich) in a 1:70 ratio (TEV:Protein) overnight at room temperature. Following cleavage, the solution was applied to a Ni-NTA-agarose affinity resin and the flow through containing cleaved protein was collected. Proteins were dialyzed into 50 mM PB (pH 7.2) and stored at 4 °C. Finally, proteins were purified by size exclusion chromatography using a Superose 6 GL 10/300 gel filtration column for ODAM or a S200 gel filtration column for AMTN (GE Healthcare). Columns were pre-equilibrated with 50 mM PB (pH 7.4). Fractions were evaluated for purity by SDS-PAGE. SCPPPQ1 was expressed in pHT*hScpppq1* and grown and purified in the same conditions than ODAM and AMTN but in denaturing conditions where buffers contained 8 M of Urea.

### Circular Dichroism

Solutions of purified complex were prepared at 10, 5, 2.5, and 1.25 μM in 50 mM PB (pH 7.2). CD measurements were performed using cuvettes with a path length of 0.1 cm (far-UV CD, 180–250 nm). The far-UV CD spectra were collected using a Jasco J-810 spectropolarimeter between 180–250 nm wavelengths with a data pitch of 0.5 nm, a bandwidth of 1 nm, a scan speed of 50 nm/min, and a response time of 1 min. All spectra were corrected for buffer contribution. Each spectrum is an average of 3 scans. MRW was calculated by assuming an average mean residue weight of 110 Daltons for protein mixtures. Analysis of the CD spectra was carried out using the CDSSTR method from the dichroweb server (http://dichroweb.cryst.bbk.ac.uk) for analyzing protein CD spectra for secondary structure estimation. The CD spectrum of the protein analyzed was removed from the reference set and the secondary structure fractions were determined using a reference set of 12 members.

### Atomic Force Microscopy

Atomic Force Microscopy (AFM) imaging was performed using a JEOL JSPM-5200 Scanning Probe Microscope. A 10 μl drop of sample solution (from 1 μg/ml to 100ng/ml) was incubated for 5 min on a Highly Ordered Pyrolytic Graphic (HOPG) substrate. Then, the HOPG surface was rinsed 3 times with 50 ml of distilled water (dH_2_O) and air-dried. All imaging was observed under dry conditions and was carried out using the tapping mode at room temperature. The cantilevers used (HQ-NSC14, MicroMasch, USA) had a spring constant of 5.7 N/m. The scan speed was 0.5 Hz and scan size was 1.5 × 1.5 mm. The samples were visualized as topographic images and 3D images at a resolution of 512 × 512 pixels. Because recombinant SCPPPQ1 readily precipitates during purification in the neutral buffer used for AMTN and ODAM or under other non-denaturing buffers, it was purified under denaturing conditions with 8 M of urea. For imaging SCPPPQ1 by AFM, the proteins were solubilized in buffer containing urea concentrations in very low concentration of urea (100 mM) before to be incubated on HOPG.

### Transmission Electron Microscopy

Specimens were prepared for EM using a conventional negative staining procedure. A 5 μl drop of purified proteins was adsorbed for 2 min to a glow-discharged carbon-coated copper grid and stained twice for 1 min with freshly prepared 2% uranyl acetate in dH_2_O. Samples were imaged using a FEI Tecnai T12 (Eindhoven, The Netherlands) Transmission Electron Microscope (TEM) equipped with a LaB6 filament and operated at an acceleration voltage of 80 kV. Some specimens were also prepared for cryo-EM. 3 μl of solution were deposited on glow-discharged quantifoil R 2/2 grids (Quantifoil Micro Tools GmbH, Jena, Germany), blotted 2 s, and flash frozen in liquid ethane. Cryo-samples were examined with a Titan Kryos (Eindhoven, The Netherlands) operating at 300 kV.

## Additional Information

**How to cite this article**: Fouillen, A. *et al*. Interactions of AMTN, ODAM and SCPPPQ1 proteins of a specialized basal lamina that attaches epithelial cells to tooth mineral. *Sci. Rep.* **7**, 46683; doi: 10.1038/srep46683 (2017).

**Publisher's note:** Springer Nature remains neutral with regard to jurisdictional claims in published maps and institutional affiliations.

## Supplementary Material

Supplementary Figures and Tables
