# Supplementary material for: Interactions of AMTN, ODAM and SCPPPQ1 proteins of a specialized basal lamina that attaches epithelial cells to tooth mineral
Source: Sci Rep. 2017 Apr 24;7:46683. doi: 10.1038/srep46683 (PMC5402393; doi:10.1038/srep46683)
Supplement: Supplementary Figures and Tables [file srep46683-s1.pdf]

Interactions of AMTN, ODAM and SCPPPQ1 proteins of a specialized basal lamina that attaches epithelial cells to tooth mineral

Aurélien Fouillen[1,2], Juliana Dos Santos Neves[1], Charline Mary[2], Jean-Daniel Castonguay[1], Pierre Moffatt[3], Christian Baron[2], Antonio Nanci[1,2*]

[1] Laboratory for the Study of Calcified Tissues and Biomaterials, Department of Stomatology, Faculty of Dentistry, and [2] Department of Biochemistry and Molecular Medicine, Faculty of Medicine, Université de Montréal, Montréal, Québec, Canada.
[3] Shriners Hospital for Children, Montréal, Québec, Canada.

*Corresponding author: Antonio Nanci
Laboratory for the Study of Calcified Tissues and Biomaterials,
Department of Stomatology, Faculty of Dentistry,
Université de Montréal, Montréal, Québec, Canada
e-mail: antonio.nanci@umontreal.ca

Fig S1

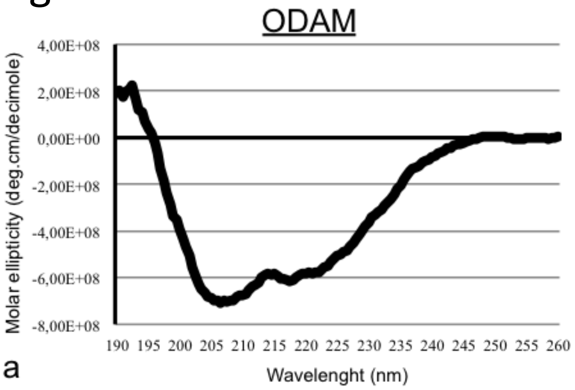

a

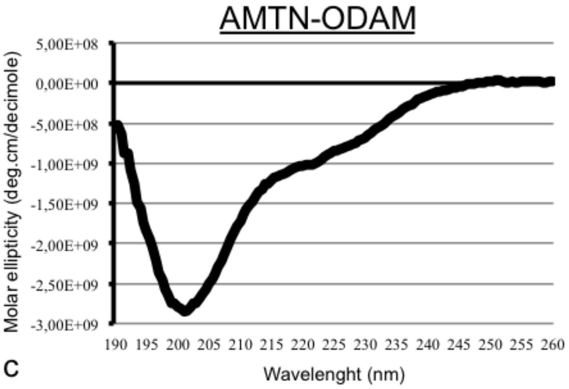

c

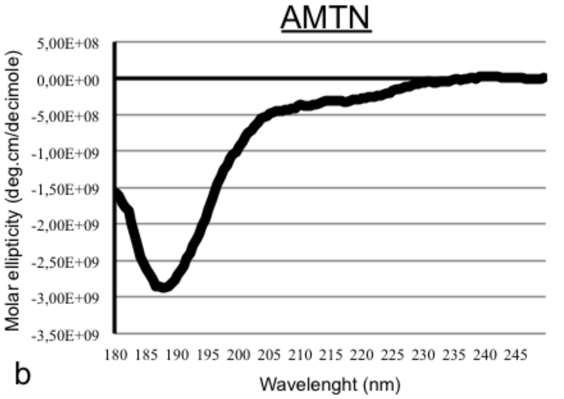

b

d	ODAM	AMTN	ODAM-AMTN
Helix 1	0	0.01	0.38
Helix 2	0.03	0.03	0.16
Strand 1	0.23	0.26	0.10
Strand 2	0.12	0.14	0.08
Turns	0.13	0.12	0.10
Unordered	0.47	0.44	0.18
Total	1	1	1
NMRSD	0.08	0.059	0.001
MRW	111.5	103.34	107.5

Fig S2

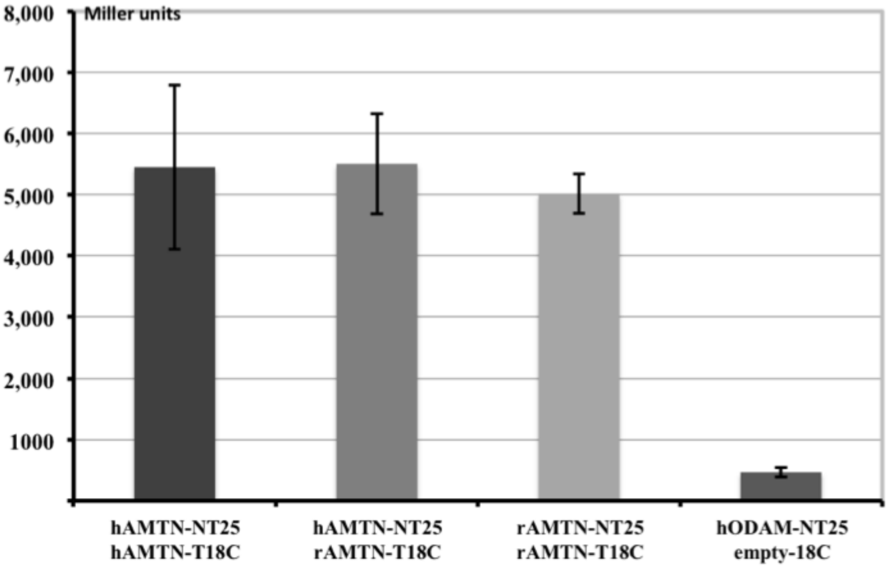

Miller units
8,000
7,000
6,000
5,000
4,000
3,000
2,000
1000
hAMTN-NT25
hAMTN-T18C
hAMTN-NT25
rAMTN-T18C
rAMTN-NT25
rAMTN-T18C
hODAM-NT25
empty-18C

Fig S3

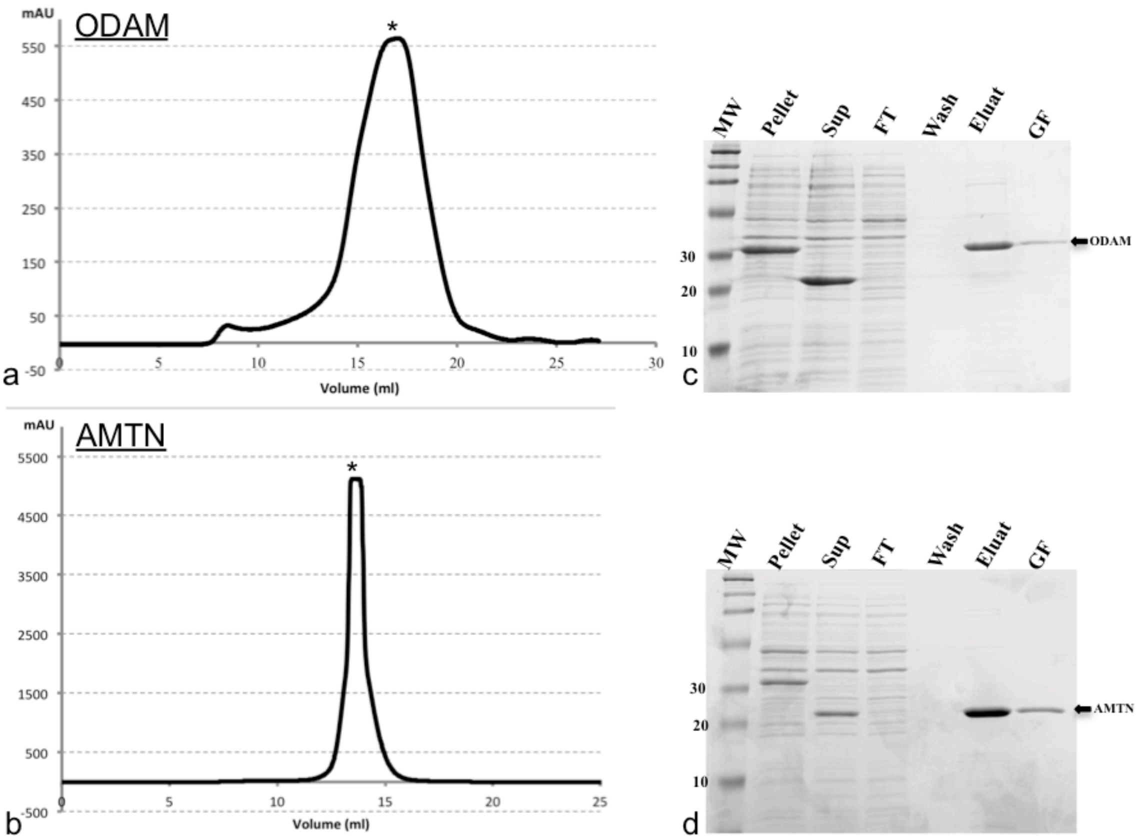

ODAM
mAU
550
450
350
250
150
50
-50
0
5
10
15
20
25
30
Volume (ml)
a
AMTN
mAU
5500
4500
3500
2500
1500
500
-500
0
5
10
15
20
25
Volume (ml)
b
MW
Pellet
Sup
FT
Wash
Eluat
GF
30
20
10
ODAM
c
MW
Pellet
Sup
FT
Wash
Eluat
GF
30
20
10
AMTN
d

Table S4 : Primers and restriction site for cloning procedures

Protein	Strain	Direction	Primer	Restriction site
**human ODAM**	pHT	Forward	5’ CGG**GGTACC**ATGGCCCCACTTATCCCAC 3’	KpnI
	pHT	Reverse	5’ CGC**GGATCC**TTACGGTTCCCTTAGGCTGTC 3’	BamHI
	PUT18 - PKNT25	Forward	5’ CGC**GGATCC**CATGAAAATTATAATTCTT 3’	BamHI
	PUT18 - PKNT25	Reverse	5’ CGG**GGTACC**GGTTCCCTTAGGCTGTCAG 3’	KpnI
	PUT18C - PKT25	Forward	5’ CGC**GGATCC**CATGAAAATTATAATTCTT 3’	BamHI
	PUT18C - PKT25	Reverse	5’ CGG**GGTACC**TATGGTTCCCTTAGGCTGT 3’	KpnI
**rat ODAM**	pHT	Forward	5’ CGG**GGTACC**ATGAGCAACAGCCATGAGT 3’	KpnI
	pHT	Reverse	5’ CGC**GGATCC**TTATGGTTCTCTTAGGCTATC 3’	BamHI
	PUT18 - PKNT25	Forward	5’ CGC**GGATCC**CATGAAAATTATAATTCTT 3’	BamHI
	PUT18 - PKNT25	Reverse	5’ CGG**GGTACC**CCTGGTTCTCTTAGGCTAT 3’	KpnI
	PUT18C - PKT25	Forward	5’ CGC**GGATCC**CATGAAAATTATAATTCTT 3’	BamHI
	PUT18C - PKT25	Reverse	5’ CGG**GGTACC**TTATGGTTCTCTTAGGCTA 3’	KpnI
**human AMTN**	pHT	Forward	5’ CGC**GGATCC**ATGTTACCACAGCTCAAA 3'	BamHI
	pHT	Reverse	5’ CCC**AAGCTT**TTACTGAATTCCATTTGCTG 3’	HindIII
	PUT18 - PKNT25	Forward	5’ GC**TCTAGA**CATGAGGAGTACGATTCTA 3’	XbaI
	PUT18 - PKNT25	Reverse	5’ CGC**GGATCC**CGCTGAATTCCATTTGCTG 3’	BamHI
	PUT18C - PKT25	Forward	5’ GC**TCTAGA**CATGAGGAGTACGATTCTA 3’	XbaI
	PUT18C - PKT25	Reverse	5’ CGC**GGATCC**TTACTGAATTCCATTTGCT 3’	BamHI
**rat AMTN**	pHT	Forward	5’ CGG**GGTACC**ATGTTGCCAAGGCAGCT 3'	KpnI
	pHT	Reverse	5’ GC**GGATCC**TTACTTAGTTCTATTTGGTGGGT 3’	BamHI
	PUT18 - PKNT25	Forward	5’ GC**TCTAGA**GATGAAGACCGTGGTTCTC 3’	XbaI
	PUT18 - PKNT25	Reverse	5’ CGC**GGATCC**TTAGTTCTATTTGGTGGGT 3’	BamHI
	PUT18C - PKT25	Forward	5’ GC**TCTAGA**GATGAAGACCGTGGTTCTC 3’	XbaI
	PUT18C - PKT25	Reverse	5’ CGC**GGATCC**CTATTTAGTTCTATTTGGT 3’	BamHI
**human SCPPPQ1**	pHT	Forward	5’ CGG**GGTACC**ATGCTTGGACAATCTGGAGG 3'	KpnI
	pHT	Reverse	5’ CGC**GGATCC**TTATCTCCCAAGGAAGCCC 3’	BamHI
	PUT18 - PKNT25	Forward	5’ CCC**AAGCTT**CATGCTTGGACAATCTGGAGG 3’	HindIII
	PUT18 - PKNT25	Reverse	5’ CG**GGATCC**CGTCTCCCAAGGAAGCCCT 3’	BamHI
	PUT18C - PKT25	Forward	5’ GC**TCTAGA**CATGGCTCTGCCCATCCCC 3’	XbaI
	PUT18C - PKT25	Reverse	5’ CG**GGATCC**TTATGTCCCAGAGAAGTTCCC 3’	BamHI
**rat SCPPPQ1**	PUT18 - PKNT25	Forward	5’ GC**TCTAGA**CATGCTTGGACAATCTGGAGGG 3'	XbaI
	PUT18 - PKNT25	Reverse	5’ CG**GGATCC**TTATCTCCCAAGGAAGCCC 3’	BamHI
	PUT18C - PKT25	Forward	5’ GC**TCTAGA**CATGCTTGGACAATCTGGA 3’	XbaI
	PUT18C - PKT25	Reverse	5’ CG**GGATCC**TTATCTCCCAAGGAAGCCC 3’	BamHI

Supplementary legends:

Fig S1: **Structural analysis by circular dichroïsm spectroscopy**. Spectra obtained for **(A)** ODAM, **(B)** AMTN, and **(C)** the mix of ODAM and AMTN. **(D)** Table of calculated secondary structure.

Fig S2: **Interspecies bacterial two-hybrid interaction analysis.** Here illustrated for AMTN**,** no significant difference in interaction capacity between the rat and human forms was noted.

Fig S3: **Analysis of expression of hODAM and hAMTN by *E. coli***. Size exclusion chromatography of **(A)** hODAM and **(B)** hAMTN results in single distinct peaks. The hatched lines denote the fractions used for **(C-D)** SDS-PAGE gels analysis to evaluate purity of the eluted proteins. MW= Molecular weight
